# Transferrin Insufficiency and Iron Overload in Follicular Fluid Contribute to Oocyte Dysmaturity in Infertile Women With Advanced Endometriosis

**DOI:** 10.3389/fendo.2020.00391

**Published:** 2020-06-19

**Authors:** Anji Li, Zhexin Ni, Jie Zhang, Zailong Cai, Yanping Kuang, Chaoqin Yu

**Affiliations:** ^1^The First Affiliated Hospital of Guizhou University of Traditional Chinese Medicine, Guiyang, China; ^2^Department of Gynecology of Traditional Chinese Medicine, Changhai Hospital, Naval Medical University, Shanghai, China; ^3^Department of Assisted Reproduction of the Ninth People's Hospital, Shanghai, China; ^4^Department of Biochemistry and Molecular Biology, Naval Medical University, Shanghai, China

**Keywords:** endometriosis, infertility, follicular fluid, transferrin, oocyte

## Abstract

**Purpose:** To screen out specific protein with different concentration in follicular fluid from advanced endometriosis and determine its direct effect on mouse oocytes matured *in vitro*.

**Methods:** FF samples were obtained from 25 patients (EMS group, *n* = 15; control group, *n* = 10) to screen the differential proteins by using iTRAQ Labeling and 2D LC-MS. Transferrin (TRF) in was found significantly decreased in EMS group, which was verified using ELISA in enlarged FF samples (EMS group, *n* = 31; control group, *n* = 27). The contents of ferric ion in FFs were detected by ELISA and TRF saturations were calculated in two groups. Germinal vesicle (GV) oocytes of mouse were maturated *in vitro* interfered with the FFs in five groups, whose concentrations of TRF were modulated, and maturation *in vitro* rates were compared among groups.

**Results:** The reduced concentration of TRF with three analogs and increased concentration of ferric ion were found in the FF of the EMS group (*p* < 0.05). The numerical values of TSAT was 54.8% in EMS group, indicating iron overload in the FF. The EMS-FF showed significantly decreased maturation *in vitro* rate (*p* < 0.05) of mouse oocytes, which was improved with the supplementation of TRF, compared with the control-FF. The effect was blocked by the TRF antibody (*p* < 0.05).

**Conclusions:** Being aware of the relatively small sample size, our results possibly suggest that TRF insufficiency and iron overload in FF from advanced EMS contribute to oocytes dysmaturity, which may be a cause of EMS-related infertility.

## Introduction

Endometriosis (EMS) is an estrogen (E2)-dependent disease wherein the endometrial stromal and glandular epithelial cells are externally implanted from the uterus. The incidence rate is ~2–10% (1), and 20–50% of women can be infertile (2). At present, the changes in the reproductive tract anatomy, impaired follicular development, ovulation dysfunction, embryo implantation difficulties, and other factors may cause infertility in patients with EMS (3).

Assisted reproductive technology (ART) is a common choice for women with EMS-associated infertility to achieve pregnancy (4). However, patients with EMS have less oocytes retrieved and higher rate of cycle cancellation than EMS-free controls in oocytes retrieval cycles (5–7). The number of oocytes retrieved directly reflects ovarian response and is one of the best clinical markers of oocyte quality, which indicates their ability to complete maturation and undergo successful fertilization (8). The decreased number of mature oocytes retrieved in women with endometriosis compared to women with other causes of infertility was reported in prospective case-control studies (8–10) and meta-analysis (11), which indicated dysmaturity of oocytes of patients with EMS. However, the mechanism of dysmaturity of oocytes in endometriosis remain largely unknown.

Follicular fluid (FF) is secreted by the ovarian granulosa cells and disseminated by serum. Moreover, FF constitutes the microenvironment for the growth and development of oocytes before ovulation. Abnormalities of EMS FF have been widely confirmed. Karaer et al. (12) performed nuclear magnetic resonance spectroscopy analysis on EMS FF and analyzed the metabolic composition of FF with univariate and multivariate statistical analysis of nuclear magnetic resonance data. The results showed that lactic acid, B-qlucose, and pyruvate and valine content in FF of ovarian EMS increased significantly. Another study has found that differential metabolites in the FF of patients with severe EMS-related infertility involve cell proliferation and apoptosis, energy metabolism, inflammatory response, and angiogenesis (13). Some certain molecules were observed, whose changes in levels in the FF of patients with EMS (14–16) was closely correlated with the stages of EMS according to the American Society for Reproductive Medicine (17).

The FF-related oocyte dysmaturity is hypothesized to be an important cause of EMS-associated infertility. The level of oxidative stress in EMS FF is increased (18, 19) and the function of differentially expressed protein molecules in EMS FF is mainly concentrated in the positive regulation function of the response to reactive oxygen species (20). Our previous research results show that using EMS FF to interfere with the *in vitro* maturation of mouse oocytes can increase the level of oxidative stress and reduce the maturation rate in oocytes (21). It is suggested that the increase of reactive oxygen species in FF is related to the poor quality of oocytes and embryos (22).

However, the link between their levels and their influences on oocytes quality were not completely established because of the complexity of FF. Therefore, the aims of this study are (i) to screen out the specific protein, whose concentration changes significantly in FF from advanced EMS; (ii) to explore the mechanism of the change of the specific protein; (iii) to investigate the direct effect of the specific protein on mouse oocytes maturation *in vitro*.

## Materials and Methods

### Patients and FF Sampling

A total of 58 women who underwent ART from January 1, 2017 to December 31, 2017 at Department of Assisted Reproduction of the Ninth People's Hospital in Shanghai were included in this study. The study protocol was approved by the Ethics Committee (Institutional Review Board) of the Ninth People's Hospital. All participants provided informed consent before counseling for infertility treatments and routine ART procedures.

Patients with stages III–IV EMS according to the revised American Fertility Society classification were selected. The eligibility criteria were as follows: 25–40 years old, body mass index (BMI) of 18.5–23.9 kg/m2, first IVF, and normal sperm for males. The control group consisted of women with infertility caused by tubal factors, including bilateral salpingemphraxis and tubal resection. Controlled ovarian hyperstimulation to patients was performed by 150–225 IU/day of human menopausal gonadotropin (hMG) (Anhui Fengyuan Pharmaceutical Co., China) and 4 or 10 mg/day of medroxyprogesterone acetate (MPA) (Beijing ZhongXin Pharmaceutical, China) from menstrual cycle day 3, according to the observed follicular growth by ultrasound and blood test. Ovulation was triggered by human chorionic gonadotropin (hCG) 2,000–10,000 IU (Lizhu Pharmaceutical Trading Co., China) when there were more than three dominant follicles >18 mm in diameter, followed by transvaginal ultrasound-guided oocyte retrieval 36–37 h later. No difference was found between the two groups in terms of age, BMI and duration, and dose of human menopausal gonadotropin and medroxyprogesterone acetate.

FF was aspirated in individual sterile tubes when the mature follicles were punctured. Only FF free from blood contamination upon visual inspection was used. Samples were centrifuged at 1,000 rpm for 10 min to separate cell remnants, and the supernatant was stored at −40°C for further use.

### iTRAQ Labeling and 2D LC–MS

Five individual FF samples were randomly selected and collected from five patients, and a pooled FF sample was composed for iTRAQ test to reduce individual differences in samples. Three pooled FF samples composed of 15 individuals in the EMS group and two pooled FF samples composed of 10 individuals in the control group were selected. Before the mixing process, the total protein concentration of each individual FF sample was detected using the bicinchoninic acid assay (23). Sodium dodecyl sulfate–polyacrylamide gel electrophoresis and principal component analysis were performed to detect the protein distribution in each pooled sample and the sample repeatability, respectively, for each group (24). Three pooled samples in the EMS group were then labeled with the iTRAQ® reagents 113, 114, 115, and two pooled samples in the control group were labeled with the iTRAQ® reagents 119, 121, following manufacturer's instructions (Applied Biosystems). The next steps in protein processing was referred to the previously described literature (25, 26).

Data were processed employing the Protein Pilot Software v. 5.0 (AB SCIEX, USA) against the Homo sapiens database by using the Paragon algorithm (27). The experimental data from LC–MS was used to match the theory data to obtain the result of protein identification. Proteins were identified using the search option: emphasis on biological modifications. For the iTRAQ analysis, fold changes (FC) were adopted to compare the protein differences between the EMS and control groups. The *T*-test was used to analyze the *p*. The protein with the FC cutoff ratio of more than 1.2 or <0.8, as well as a *p* < 0.05, was designated as differential protein expression between two groups.

### Detection of TRF and Ferric ion and Calculation of TRF Saturation (TSTA) in FF

ELISA was performed on the TRF between the two groups at a larger sample (FF from 31 patients in EMS group and 27 patients in control group) to confirm the iTRAQ results. The concentration of ferric ion in FF was also detected by ELISA, which was conducted in accordance with the manufacturer's instructions (Enzyme-linked Biotechnology Co., Ltd., Shanghai, China).

The total iron-binding capacity (TIBC) is the total amount of non-specific binding of various proteins to ferric ion and can be calculated using the formula: TIBC (μmol/L) = [TRF (g/L) + 0.016]/0.047 (28). TRF saturation(TSTA) reflects the balance between ferric ion and TRF, and can be calculated as follows: TSTA (%) = ferric ion (μmol/L)/TIBC (29). More than 45% TSTA indicates iron overload (30), and more than 80–85% of the highly toxic non-TRF-bound iron can cause organ damage (31, 32).

### Effect of TRF in FF on Mouse Oocyte Maturation *in vitro*

Mouse oocytes that matured *in vitro* in this study were from female Kun Ming Bai mice from the SLAC Laboratory Animal Co. Ltd (Shanghai, PRC). All mice were maintained under specific pathogen-free conditions and given sterilized water and fodder at 20°C and 40% humidity in the Experimental Animal Center of the Ninth People's Hospital. The mice (3–4 weeks of age) were injected with 7.5 IU pregnant mare serum gonadotropin (Tianjin Animal Hormone Factory, Tianjin City, China) and then sacrificed by cervical dislocation after 46–48 h.

The oocytes with a germinal vesicle (GV) were collected in preheated human tubal fluid (HTF) medium (Millipore, Billerica, MA, USA) after laparotomy and bruising of ovaries, followed by washing thrice with fresh HTF medium without remnants. GV oocytes were cultured in droplets with different culture medium, including control FF (control group), EMS FF (EMS group), EMS FF + TRF (TRF group), EMS FF + TRF + antibody for TRF (AB group) and EMS FF + TRF + isotype control antibody (ISO group). The details of the culture medium of five groups are presented in Supplemental Table 1. All oocytes were observed using phase-contrast microscopy to distinguish different stages (Supplemental Figure 1). Since nuclear maturation indicated by germinal vesicle breakdown (GVBD) occurs before cytoplasmic maturation which produces the first polar body, oocytes matured *in vitro* were classified mainly as being in GV, GVBD, and MII. PA represents two cells with same size or a second polar body without any fertilization, which was counted out in this study. Oocyte maturation rate was calculated as the number of meiosis II (MII) oocytes divided by the total number of oocytes cultured, excluding parthenogenetic activation (PA).

### Statistical Analysis

Statistical analyses were conducted using the IBM SPSS 21.0 software (IBM, NY, USA). The Kolmogorov–Smirnov test was performed to assess the normality of distribution in continuous data, which were presented as mean ± standard deviation and assessed using the Mann–Whitney *U*-test for the two independent groups. Count data were presented as numbers and percentages and assessed using the Pearson's chi square test or the Fisher's exact test. Two-sided *p* < 0.05 was considered statistically significant for baseline data between two groups. Binary logistic regression analysis was performed with clinical pregnancy as the dependent variable, and all baseline characteristics of the patients served as independent variables. Univariate analysis was employed to avoid interference between parameters.

## Results

### Proteins in FF Identified by iTRAQ

**Figure 1:**
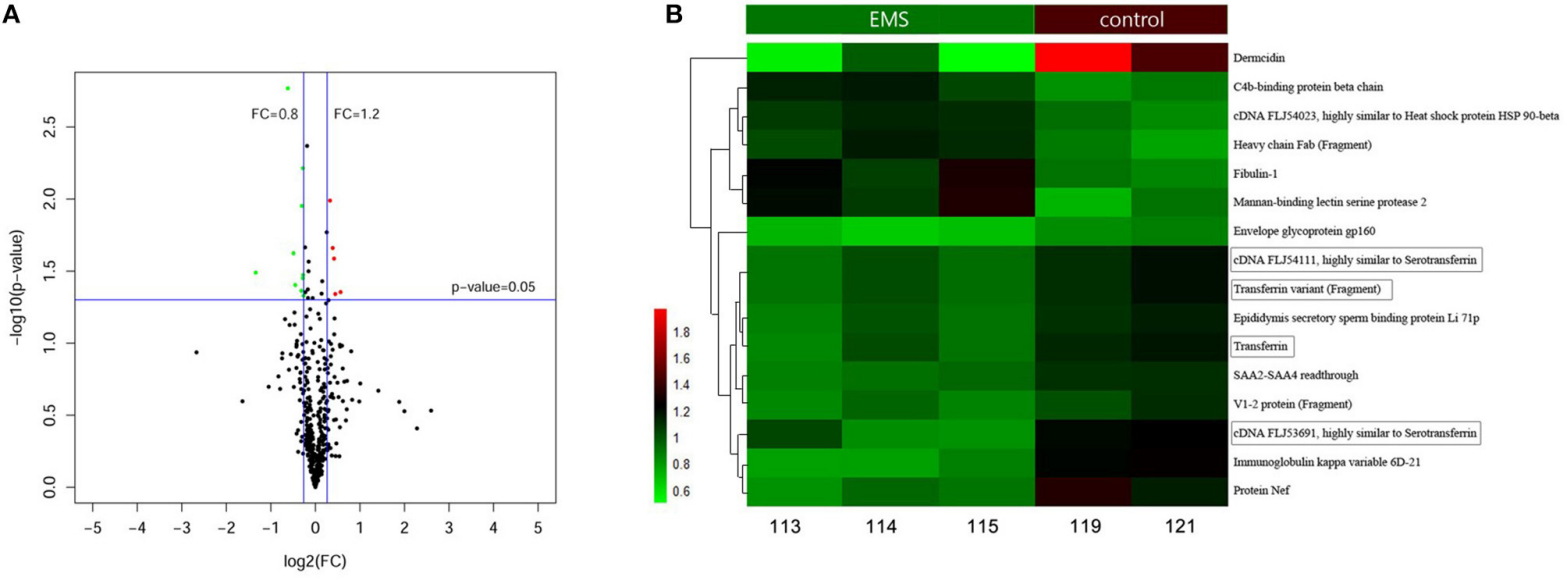
Proteins in FF identified by iTRAQ. **(A)** Volcano Plot of 474 authentic proteins expressed differently between two groups. Green signifies significantly downregulated proteins and red signifies significantly upregulated proteins in the EMS group compared with the control group. FC, fold changes. **(B)** Hierarchical cluster analysis of 16 significant differential proteins between the EMS group (113, 114, 115) and the control group (119, 121) (*p* < 0.05).

A total of 577 proteins were identified from 20,718 distinct peptides by using the iTRAQ technique (Data Sheet 1). According to the confidence of the peptides, 474 proteins were considered authentic in the FF of the EMS and the control groups (Figure 1A, Data Sheet 1). The identified proteins were filtered using the selected filter exclusion parameters (FC >1.20 or <0.80). A total of 108 proteins from the EMS group were screened as different proteins compared with the controls (Supplemental Figure 2, Data Sheet 1), and 16 of them were considered as significant (*p* < 0.05) (Figure 1B, Data Sheet 1). The TRF with three analogs from the significant proteins [cDNA FLJ53691, cDNA FLJ54111, TRF variant (Fragment)] were all downregulated in the EMS group. Thus, TRF was given focus in the following experiments.

### Confirmation of TRF and Detection of Ferric ion in FF

**Figure 2:**
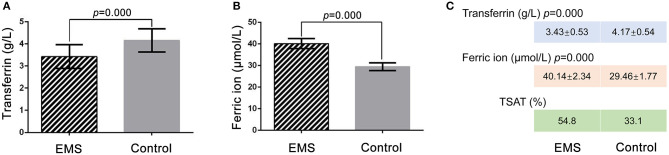
Detection of TRF and ferric ion by ELISA and TSTA in FF. **(A)** Concentration of transferrin in FF by ELISA between two groups. **(B)** Concentration of ferric ion in FF by ELISA between two groups. **(C)** Numerical value of transferrin, ferric ion and TSAT between two groups. TSTA, transferrin saturation.

ELISA was performed to confirm the TRF level in the FF of the EMS and the control groups. The TRF concentrations of FF samples were 3.43 ± 0.53 g/L (*n* = 31) in the EMS group and 4.17 ± 0.54 g/L (*n* = 27) in the control group (*p* = 0.000; Figure 2A). The ELISA results for TRF were basically consistent with those of iTRAQ. The ferric ion concentrations in FF were 40.14 ± 2.34 μmol/L (*n* = 31) in the EMS group and 29.46 ± 1.77 μmol/L (*n* = 27) in the control group (*p* = 0.000; Figure 2B). The numerical values of TSAT were 54.8 and 33.1% in the EMS and control groups, respectively (Figure 2C). These results indicated iron overload in the FF of the EMS group.

### TRF in FF Affects Mouse Oocyte Maturation *in vitro*

**Figure 3:**
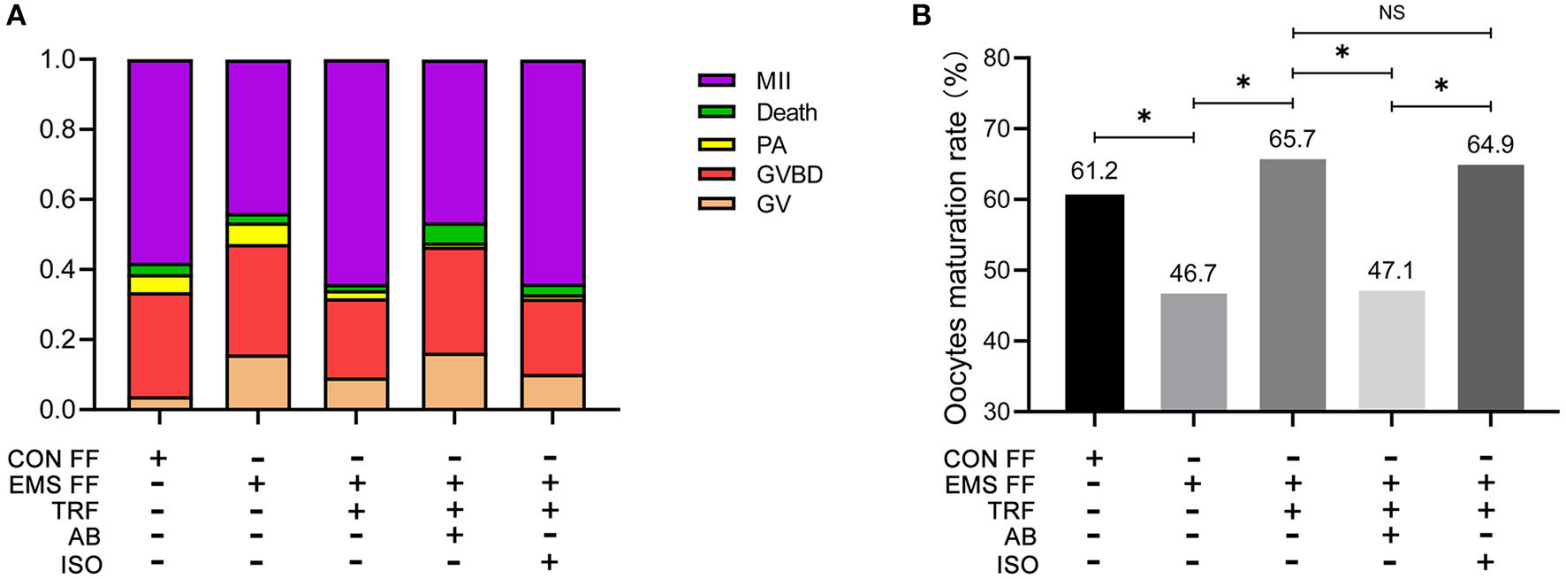
Mouse oocytes matured *in vitro* with different follicular fluid. **(A)** The proportion of different stages of oocytes cultured *in vitro* in five groups. **(B)** Comparisons of oocytes maturation rate among five groups. TRF, transferrin; FF, follicular fluid; EMS, endometriosis; TSAT, transferrin saturation; CON, control; AB, antibody; ISO, isotype control antibody; MII, Meiosis II, oocytes with complete first polar body; Death, dead oocytes; PA, parthenogenetic activation; GVBD, oocytes without germinal vesicle or first polar body; GV, oocytes with a germinal vesicle; *p < 0.05; NS, no significant difference.

As determined by phase-contrast microscopy, the numbers of oocytes in stages of GV, GV breakdown, PA, MII, and death were counted (Table 1), and the proportions of each stage were counted in the five groups (Figure 3A). The percentage of MII oocytes was considered as the maturation rate and the pairwise comparisons of maturation rate were performed in the five groups (Figure 3B). The maturation rate in the EMS FF group (46.7%) was significantly lower than that in the CON FF group (61.2%, *p* < 0.05), which was significantly reversed by the EMS FF + TRF group (65.7%) (*p* < 0.05). Furthermore, when the TRF was blocked by its antibody, the maturation rate decreased in the EMS FF group (47.1%). However, the isotype control antibody showed no effects.

**Table 1:** Stages of IVM and percentage of MII oocytes among five groups.

**Group**	**Total number of oocytes cultured**	**GV**	**GVBD**	**PA**	**Death**	**MII**	**Oocytes maturation%**
CON FF	155	6	46	8	5	90	61.2
EMS FF	146	23	46	9	4	64	46.7
EMS FF +TRF	173	16	39	4	3	111	65.7
EMS FF +TRF +AB	86	14	26	1	5	40	47.1
EMS FF +TRF +ISO	167	17	36	2	5	107	64.9

*IVM, maturation in vitro; CON, control; FF, follicular fluid; EMS, endometriosis; TRF, transferrin; AB, antibody; ISO, isotype control antibody; GV, oocytes with a germinal vesicle; GVBD, oocytes with germinal vesicle breakdown; PA, parthenogenetic activation; Death, dead oocytes; MII, Meiosis II*.

## Discussion

FF constitutes the microenvironment for folliculogenesis and development before ovulation. The abnormalities in proteomic and metabolomic of FF from EMS have been widely confirmed. However, because of the complex composition of FF, previous studies had not pinpointed the key proteins whose concentration was associated with follicular development and oocytes competence. To our knowledge, this is the first study that focus on exact protein, whose influence on oocytes maturation was directly assessed by modulating its concentration in FF from EMS.

High-throughput screening was performed using iTRAQ and further validated using ELISA with enlarged samples. The TRF concentration was significantly decreased in the FF of infertile patients with advanced EMS. The human TRF, an iron-carrier protein, is a 76 kDa glycoprotein that is mainly produced in the liver. Iron is essential in cell proliferation and DNA synthesis (33, 34), but mediates the production of highly toxic reactive oxygen species (ROS). Therefore, transferrin was developed to carry iron safely in the circulation (35), and transferrin saturation (TSTA) is a measure of transferrin carrying ferric ion. Cells efficiently absorb serum iron through the TRF/TRFR1 (TRF receptor 1) system, after which TRFR1 and TRF are reused. Most TRF in FF originates from the peripheral circulation and enters via endocytosis by granulosa cells, and a small part has been produced by these cells (36). Ducolomb et al. (37) uncovered that F1, which is a special protein complex-containing TRF isolated from porcine FF, can increase the proportion of mature and fertilized oocytes. Insulin–TRF–selenium has been routinely used in IVM systems for oocytes of several species, such as mice, bovine and pigs in pre-antral follicle culture systems (38–40). However, Guimaraes et al. could not detect any beneficial effect of ITS in pre-maturation medium (41).

The numerical values of concentration of TRF and ferric ion were detected by ELISA and TSAT was calculated, which revealed the iron overload in the FF of infertile women with advanced EMS.

Iron overload causes the imbalance in cell processes, cell dysfunction, and cell apoptosis or necrosis, crude lipid peroxidation, proteins, and DNA damage (42, 43). TRF insufficiency can induce the excess of ROS (44), promoting chromosome instability, and causing the difficult formation of spindle at the early stage of meiosis (45), which was already found in the FF of patients with EMS (18). In addition, EMS FF can increase the level of oxidative stress in oocytes and reduce the maturation rate *in vitro*, which was observed in our previous studies (21). ROS Granulosa cells can produce TRF and absorb TRF from peripheral circulation by endocytosis (36) and may be damaged by iron overload to influence TRF level in FF.

Although TRF insufficiency and iron overload were found in the FF of patients with advanced EMS in our study, the original reason remains unknown. Interestingly, Sanchez et al. (46) had paid attention to the levels of two types of ferritin and TRFR1 in FF and uncovered the correlation between them and the number of oocytes retrieved and embryo quality in women with endometrioma. The authors demonstrated that the level of total iron in endometrioma-proximal follicles are higher than that in endometrioma-distal ones. Half of the iron in the human body is in the blood (47). Thus, the repeated bleeding of local lesions of EMS may increase iron levels in the abdominal cavity. Iron is then delivered to the granulosa cells by peripheral TRF, which can combine to cell–surface TRFR1 and trigger endocytosis. The excess of iron may be the cause of TRF insufficiency in the FF of women with advanced EMS.

We further clarified the relevance between transferrin insufficiency in EMS-FF and oocyte competence. In our previous studies, the maturation *in vitro* rate of mouse oocytes was decreased by EMS-FF compared with the control, which was verified again in this study (46.7 vs. 61.2%, *p* < 0.05). By external addition, the concentration of TRF in EMS-FF (EMS group) equivalently matched that of in the FF from tubal infertility patients (control group). We also set up an experimental group to block the function of TRF molecules with antibodies, culture mouse oocytes *in vitro*, and observe changes in mouse oocyte maturation *in vitro*. The maturation rate was significantly increased after adding TRF (i.e., TRF group, 65.7%, *p* <0.01), and the maturation rate was comparable to that of the control group. After the antibody was added to bind to TRF (i.e., AB group), the maturation rate decreased significantly to a similar level in the EMS group. These experiments from two directions verified that TRF levels in the FF of patients with severe EMS-related infertility are too low, and iron overload exists, which affects the *in vitro* maturation rate of mouse oocytes; increase the levels of FF in patients with severe EMS-related infertility; TRF levels can increase the maturation rate of mouse oocytes *in vitro*, and this promotion cannot be achieved under the condition that the molecular function of TRF is blocked.

This study does have limitations to be considered. The relevance between the level of TRF in FF and the competence of oocytes needs to be further confirmed, both of which being from the same follicle. In the patients, the types were not distinguished, such as ovarian endometrioma, superficial peritoneal endometriosis, or deep infiltrating endometriosis, which causes difficulties to analyze the generation mechanism of the abnormal compositions in EMS-FF.

Significantly different levels of interleukins, hormones, peroxides, and proteins are related to IVF outcomes in patients with EMS, but the relationship between EMS fertility and TRF in FF have never been reported. Seven biological repetitions of mouse oocyte maturation were conducted to increase the credibility of the experiment and decrease the error. Our study highlights that TRF insufficiency and iron overload in FF of advanced EMS significantly affect oocyte maturity, which may one of causes of the reduced oocytes retrieved in ART. Our study provides an insight into the drug development and treatment of infertility in women with advanced EMS.

## Data Availability Statement

The raw data supporting the conclusions of this article will be made available by the authors, without undue reservation.

## Ethics Statement

The studies involving human participants were reviewed and approved by Ethics Committee (Institutional Review Board) of the Ninth People's Hospital of Shanghai. The patients/participants provided their written informed consent to participate in this study. The animal study was reviewed and approved by Ethics Committee of Changhai Hospital.

## Author Contributions

CY, ZC, and YK supervised the entire study, including the procedures, conception, design and completion, and participated in the interpretation of the study data and in revisions to the article. JZ was responsible for the collection of follicular fluid samples. ZN participated in the organization figures and tables and part of article drafting. AL contributed all the experiments and drafted the article. All authors contributed to the article and approved the submitted version.

## Conflict of Interest

The authors declare that the research was conducted in the absence of any commercial or financial relationships that could be construed as a potential conflict of interest.
