# Supplementary figures and images for: Transferrin Insufficiency and Iron Overload in Follicular Fluid Contribute to Oocyte Dysmaturity in Infertile Women With Advanced Endometriosis
Source: Front Endocrinol (Lausanne). 2020 Jun 19;11:391. doi: 10.3389/fendo.2020.00391 (PMC7317002; doi:10.3389/fendo.2020.00391)

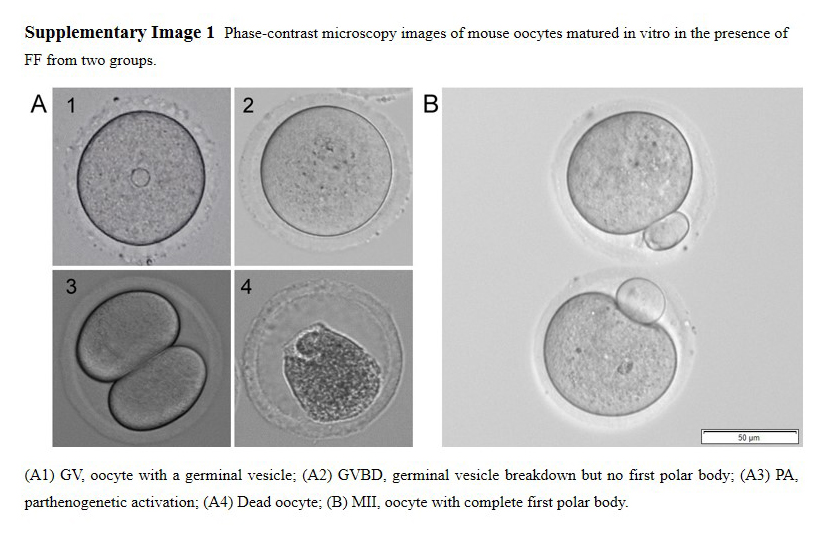

Supplement: Supplementary file 1 [file Image_1.jpg]

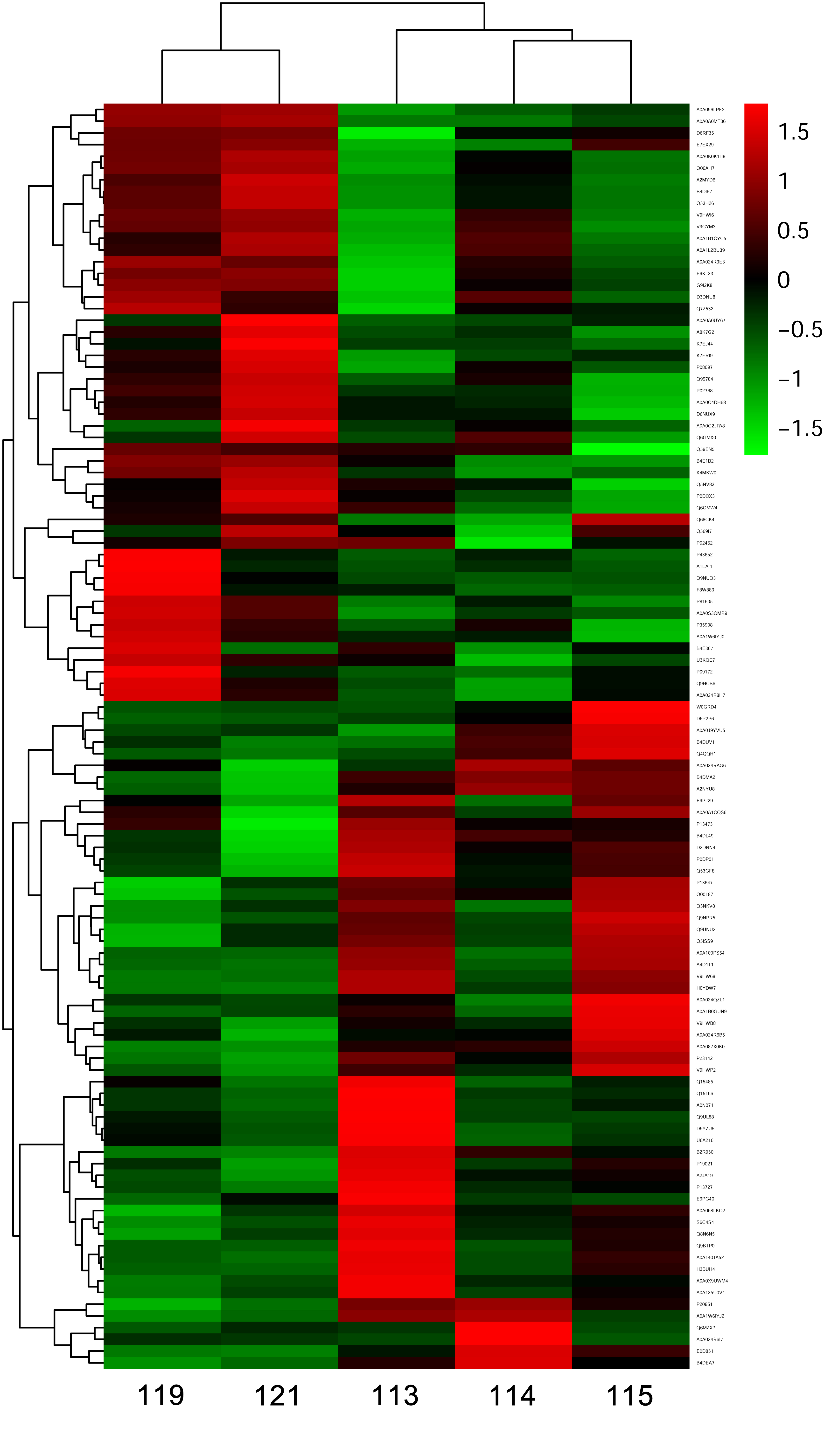

Supplement: Supplementary file 2 [file Image_2.jpg]
