# Supplementary material for: Transferrin Insufficiency and Iron Overload in Follicular Fluid Contribute to Oocyte Dysmaturity in Infertile Women With Advanced Endometriosis
Source: Front Endocrinol (Lausanne). 2020 Jun 19;11:391. doi: 10.3389/fendo.2020.00391 (PMC7317002; doi:10.3389/fendo.2020.00391)
Supplement: Supplementary file 3 [file Table_1.docx]

**Supplemental Table 1** IVM culture medium for each group

| Groups | Medium | Medium components(μL) |
| --- | --- | --- |
| Control group | CON FF | KSOM medium 320 + Control-FF 80 |
| EMS group | EMS FF | KSOM medium 320 + EMS-FF 80 |
| TRF group | EMS FF +TRF | KSOM medium 290 + EMS-FF 80 + transferrin 30(2mg/ml) |
| AB group | EMS FF +TRF +AB | KSOM medium 290（including transferrin antibody 2(890ug /ml )）+ EMS-FF 80 + transferrin 30 |
| ISO group | EMS FF +TRF +ISO | KSOM medium 290（including isotype control antibody (890ug /ml )）+ EMS-FF 80 + transferrin 30 |

IVM, maturation in vitro; CON, control; FF, follicular fluid; EMS, endometriosis. TRF, transferrin. AB, antibody; ISO, isotype control antibody; KSOM, potassium simplex optimized medium. The amount of TRF (Sigma, St. Louis, MO, USA) added into mediums was according to TRF concentration in control group detected by Elisa. The antibody for TRF (AB) (Proteintech, Rosemont, IL, USA) was added at a dilution of 1:200, which was the maximum concentration without interfering IVM results. The concentration of isotype control antibody (ISO) (Sigma, St. Louis, MO, USA) added into mediums was same as AB. The micro drops were set in tissue culture dishes (Falcon 3001; Becton Dickinson, USA) overlaid with mineral oil (Irvine Scientific, USA) at 37°C under 5% CO_2_ and 95% humidity for 16 h.
